# Predicting performance of elite kickboxers using the multi‐states theory framework

**DOI:** 10.1002/ejsc.12031

**Published:** 2024-03-05

**Authors:** Marco Morrone, Lucia Ventura, Irene Roggio, Andrea Di Blasio, Montse Ruiz, Franca Deriu, Lucia Cugusi, Andrea Manca, Claudio Robazza

**Affiliations:** ^1^ Department of Biomedical Sciences University of Sassari Sassari Italy; ^2^ Department of Medicine and Aging Sciences Behavioral Imaging and Neural Dynamics Center G. d'Annunzio University of Chieti and Pescara Chieti Italy; ^3^ Faculty of Sport and Health Sciences University of Jyväskylä Jyväskylä Finland; ^4^ Unit of Endocrinology Nutritional and Metabolic Disorders AOU Sassari Sassari Italy

**Keywords:** challenge and threat, concentration disruption, emotional arousal, MuSt theory, psychobiosocial experiences, self‐confidence, worry

## Abstract

Using the multi‐states (MuSt) theory framework, this study examined the interplay between self‐confidence, emotional arousal control, worry, concentration disruption, challenge and threat appraisals, psychobiosocial experiences, and self‐evaluated performance of medalist kickboxers involved in the WAKO World Kickboxing Championship 2021. Participants were 103 gold, silver, or bronze medalists (58 women and 45 men), aged 18–39 (*M* = 25.16 ± 4.54 years), who were contacted via email and social media and asked to fill an online survey 3 months after the event. According to the MuSt theory predictions, self‐confidence and emotional arousal control were positively related to challenge appraisal, functional psychobiosocial experiences, and self‐evaluated performance. Worry and concentration disruption were positively associated with threat appraisal, and negatively related to functional psychobiosocial experiences; concentration disruption was also negatively related to self‐evaluated performance. Results from path analysis revealed a positive indirect link from self‐confidence to self‐evaluated performance via challenge appraisal and psychobiosocial experiences. Negative indirect links from worry and concentration disruption to self‐evaluated performance through threat appraisal and psychobiosocial experiences were significant. A positive indirect effect from emotional arousal control to self‐evaluated performance via psychobiosocial experiences was also shown. The findings are discussed in light of the MuSt theory.

## Highlights

- According to the multi‐states (MuSt) theory predictions, we observed that self‐confidence and emotional arousal control were positively related to challenge appraisal, functional psychobiosocial experiences, and self‐evaluated performance.
- We also showed that worry and concentration disruption were positively associated with threat appraisal, and negatively related to functional psychobiosocial experiences, with concentration disruption being also negatively related to self‐evaluated performance.
- We provide preliminary support to the multidimensional interplay between functional (i.e., self‐confidence and emotional arousal control) and dysfunctional (i.e., worry and concentration disruption) individual dispositions, challenge and threat appraisals, psychobiosocial experiences, and performance.
- We recommend that athletes adopt self‐regulation strategies, such as self‐talk, imagery, cognitive restructuring, mindfulness, and action monitoring to improve their self‐confidence, challenge appraisal, functional emotions, and ability to manage competitive pressure.

## 1 INTRODUCTION

Athletes often report a range of subjective emotional experiences that have a significant effect on their performance in training and competition (Hanin, 2007). As an essential aspect of human adaptation, emotional experiences impact a person's effort, focus, decision‐making, memory, behavioral responses, and interactions with others (Coppin & Sander, 2021). An important goal of applied sport psychology research is the development of effective self‐regulation strategies to help athletes control and fine‐tune their emotions in order to achieve and maintain optimal levels of performance (Beatty & Janelle, 2019; Ruiz et al., 2017).

A theoretical framework developed to advance our understanding of individual experiences and their relationship with performance is the multi‐states (MuSt) theory (Ruiz, Bortoli, & Robazza, 2021). This theory offers a comprehensive and integrative framework to explain the various performance states athletes go through in training and competition. It is meant to provide a description and understanding of idiosyncratic performance experiences, predict performance, and identify the most effective strategies for the regulation of emotions and actions (Ruiz, Luojumäki, et al., 2021). In the present study, our focus was on performance predictions based on the MuSt theory, which considers performance as a dynamic and multidimensional process that involves the interactions between the individual, the task, and the environment (antecedents). This process also encompasses appraisals of perceived resources to handle task demands (mediators), emotion‐related (psychobiosocial) experiences (mediators or outcomes), and performance (outcome). Self‐confidence, emotional arousal control, worry, and concentration disruption are among the individual factors that influence pre‐competitive experiences and exert beneficial or detrimental effects on athletic performance. These factors are at the start of the process leading to performance outcome and can determine how an individual feels in a given sport situation (Ruiz & Robazza, 2020).

One of the most critical factors leading to successful performances is self‐confidence, which is defined as the belief that an athlete holds regarding their ability to achieve a particular goal or acquire the necessary physical and mental skills to express their potential (Vealey, 2007). Arousal control contributes to the management of emotions and channeling of energies toward performance, especially in high‐stress competitive situations (Baldock et al., 2021). On the other hand, worry and concentration disruption are typically regarded as detrimental for performance. Worry is conceptualized as a cognitive process characterized by doubts about one's performance relative to others and preoccupation with the repercussions of failure (Martens et al., 1990). Moreover, a high level of worry combined with a state of activation tends to cause a narrowing of attentional focus, a reduction in collection of important environmental information and, conversely, an increased perception of irrelevant stimuli (Weinberg & Gould, 2019). The ability to focus and maintain sustained attention during performance is crucial for avoiding errors and capitalizing on opponents' weaknesses by quickly identifying the most relevant information. In combat sports, for example, when an athlete fails to focus attention effectively, the relevant stimuli needed to anticipate the opponents' intentions are hardly detected and, therefore, technical and tactical performances are hampered (Sanchez‐Lopez et al., 2016).

A construct that mediates the relationship between individual dispositional characteristics, emotions, and performance is the individual perception of competition either as a challenge or as a threat. The MuSt theory, like other theoretical frameworks (Blascovich, 2008; Meijen et al., 2020), posits that these distinct patterns of cognitive evaluations influence performance. *Challenge* appraisal derives from the individual's belief of having sufficient personal resources to handle a task and viewing environmental demands as opportunities for growth, mastery, or gain. On the other hand, *threat* appraisal is elicited when individual resources are perceived as insufficient and task demands are viewed as potentially harmful. Within the MuSt theory, a challenge appraisal leads to emotion‐related (psychobiosocial) experiences that are functional for performance and involve high task engagement, while a threat appraisal leads to psychobiosocial experiences that are dysfunctional for performance and reflect low task engagement (Ruiz et al., 2023).

Psychobiosocial experiences (or states) are defined as a variety of emotional and non‐emotional manifestations of subjective feelings related to past, present, and future (anticipated) performances (Hanin, 2007). Such experiences include psychological (e.g., cognitive, confidence, motivational), biological (bodily, motor‐behavioral), and social (e.g., communicative, social support) components (modalities). A key notion of psychobiosocial experiences is functionality, which depends on an individual's perception of helpful (functional) or harmful (dysfunctional) impact of experiences on performance, availability of resources to deal with situational demands, and self‐regulation skills (for a full discussion, see Ruiz et al., 2016; Ruiz & Robazza, 2020).

How antecedents, cognitive appraisal, and psychobiosocial experiences relate to one another in a functional or dysfunctional manner for performance has been the topic of study which is gaining research attention in both team and individual sports. In carom billiard (Di Corrado et al., 2015), a positive relationship was observed between self‐efficacy (both technical and cognitive) and performance, with functional psychobiosocial states as mediating factors. Additionally, dysfunctional psychobiosocial states were negatively related to technical self‐efficacy. In ice‐hockey (Ruiz, Luojumäki, et al., 2021), the MuSt theory provided a theoretical framework for the assessment of core action elements and feeling states on performance, as well as the effectiveness of a 30‐day intervention program targeting self‐regulation. While there were no significant results in overall performance, the players perceived the intervention program as beneficial for self‐regulation. The interplay between perfectionism traits (i.e., perfectionistic strivings and concerns), cognitive appraisals, and functional/dysfunctional psychobiosocial states has also been examined within the MuSt theory framework (Ruiz et al., 2023). In line with the theoretical assumptions, challenge appraisals mediated the relationship between perfectionistic strivings and functional psychobiosocial states, while threat appraisals mediated the relationship between perfectionistic concerns and dysfunctional psychobiosocial states. These studies (Di Corrado et al., 2015; Ruiz, Luojumäki, et al., 2021, Ruiz et al., 2023) provide initial empirical evidence in support of the tenets of the MuSt theory. However, research about the interplay between individual characteristics, cognitive appraisals, psychobiosocial experiences, and performance is still scarce. Therefore, the main aim of the present study was to examine the relationship between these variables in the context of kickboxing.

Previous studies involving combat sports have investigated the effects of competition on affective states and hormonal changes (Pesce et al., 2015), while others have examined the role of self‐confidence, worry, self‐efficacy, and environmental factors on injury likelihood (Olmedilla et al., 2018). The focus of previous research was on a limited number of emotional states, such as anxiety, overlooking the diverse range of experiences athletes may have regarding their performance in competitive settings. Consequently, a more comprehensive approach could enhance our understanding of the factors leading to athletes' feeling states and their perceived performance outcomes. This approach can aid in the development of interventions aimed at enhancing performance and promoting psychological well‐being.

### 1.1 Study purpose

The purpose of the present study was to examine the relationships between self‐confidence, emotional arousal control, worry, concentration disruption, competitive appraisals, functional psychobiosocial experiences, and perceived performance of elite kickboxers. Based on assumptions outlined within the MuSt theory, we hypothesized that: (a) self‐confidence and emotional arousal control would be positively related to challenge appraisal, functional psychobiosocial experiences, and performance; and (b) worry and concentration disruption would be positively associated with threat appraisal, and negatively related to functional psychobiosocial experiences and performance (Hypothesis 1). A second aim of this study was to test whether competition appraisals and psychobiosocial states mediate the relationships between self‐confidence and emotional arousal control and perceived performance. We expected: (a) positive indirect effects from self‐confidence and emotional arousal control to performance via challenge appraisal and functional psychobiosocial experiences; and (b) negative indirect effects from worry and concentration disruption to performance through threat appraisal and psychobiosocial experiences (Hypothesis 2).

## 2 METHOD

### 2.1 Participants

The participants were selected among the medalists at the WAKO World Kickboxing Championship 2021 held in Jesolo (Italy). The inclusion criteria were as follows: (a) age between 18 and 40 years old; (b) medalists in at least one competition category; and (c) participants in ring or tatami sports, and not in musical or creative forms. Of the 258 contacted athletes who met the inclusion criteria, 103 (39.92%) agreed to participate in the study. The final sample, comprising 58 women and 45 men, aged 18–39 (*M* = 25.16 ± 4.54 years), included medalists across gold (*n* = 30), silver (*n* = 29) and bronze (*n* = 44) categories, out of a total of 394 medalists. The total number of athletes participating in the Championship was 1235 from 65 nations. Each athlete was engaged in three to five competitions during the Championship, depending on their tournament assignment and the result (i.e., win or loss). Therefore, each competition was crucial for the athlete to progress and succeed in the tournament.

### 2.2 Measures

The participants were asked to fill a multi‐section questionnaire assessing study variables.

#### 2.2.1 Sport performance psychological inventory (IPPS‐24)

The IPPS‐24 comprises the emotion higher‐order factors of the IPPS‐48 (Robazza et al., 2009), which is used to assess a range of mental skills and psychological strategies of athletes in practice and competition. The IPPS‐24 includes 24 items pertaining to four factors: Self‐confidence (e.g., “I am confident in my competitive abilities”), Emotional arousal control (e.g., “I am able to relax and control tension when needed”), Worry (e.g., “I feel panicked before competition”), and Concentration disruption (e.g., “My attention wanders while competing”). The kickboxers were asked to think about their usual competitive experiences, without referring specifically to the 2021 Championship, and to rate the frequency of the feelings and behaviors described. Items were rated on a 6‐point Likert‐type, frequency scale ranging from 1 (*never*) to 6 (*always*). Factor structure and reliability scores were acceptable, with *ω* values ranging from 0.655 (Concentration disruption) to 0.775 (Worry) in a sample of Italian athletes (Robazza et al., 2021).

#### 2.2.2 Challenge and Threat

The Challenge and Threat construal measure was used to assess the cognitive appraisals of sport competition (Adie et al., 2008). Participants were asked to respond to the 10‐item scale in relation to their actual competition during the Championship and to rate the degree in which they appraised competition in terms of a challenge (5 items; e.g., “I viewed the competition as a positive challenge”) and a threat (5 items; e.g., “I thought that the competition could be threatening to me”). Responses were rated on a 7‐point Likert‐type scale with anchors 1 (*not at all true to me*) and 7 (*very true to me*). Reliability *α* values for Challenge and Threat were 0.78 and 0.73, respectively (Adie et al., 2008).

#### 2.2.3 Psychobiosocial experience semantic differential scale in sport (PESD‐sport)

The PESD‐Sport scale (Robazza et al., 2021) was used to assess discrete emotions and performance‐related experiences in sport. This instrument includes 30 items pertaining to 10 psychobiosocial modalities (i.e., emotions, confidence, anxiety, assertiveness, cognitive, bodily‐somatic, motor‐behavioral, operational, communicative, and social support). Each item presents a negative (dysfunctional for performance) adjective on the left and its positive (functional for performance) antonym on the right of a Likert‐type scale (e.g., “unconfident–confident”, “submissive–fighting spirit”). The kickboxers were asked to rate their feelings prior to the Championship. Ratings were placed on a 9‐point, bipolar Likert‐type scale ranging from 4 (*very much*) to 0 (*neither…nor*) on the negative side and from 0 to 4 on the positive side. Scores on the dysfunctional side were then transformed into negative scores for analysis. A total score was calculated by adding the scores of the individual items. Sound factor structure and acceptable reliability, with *ω* values ranging from 0.76 (communicative modality) to 0.88 (social support modality) were found in a sample of Italian athletes (Robazza et al., 2022).

#### 2.2.4 Self‐evaluated performance

To gauge performance, five national coaches from the Italian Kickboxing Federation were asked to identify specific technical and tactical skills believed to be essential for good performance at the elite level. Each coach was asked to identify such skills independently, after which they met and discussed until reaching consensus on the skills that were best indicators of good performance and should thus be included in the questionnaire.

Seven items related to technical skills: jab and cross; hook; uppercut and back‐fist; roundhouse kick (low, middle, high); front kick and side kick; parrying, blocking, slip and weave; and footwork (stepping, half‐step, pivoting). Other four items were related to tactical skills: attack work; defense work; time management; and match setting. Guidelines for constructing efficacy measures in sport were considered (Feltz et al., 2008). The participants were asked to think about their performance during the Championship and to rate each item on an 11‐point Likert scale anchored by 1 (*extremely poor*) and 11 (*excellent*). A total score was calculated by summing the scores of the individual items.

### 2.3 Procedure

The study was conducted in accordance with the Declaration of Helsinki and was approved by the local ethical committee. The multi‐section questionnaire was constructed in an online platform (https://www.jotform.com) easily accessible through computer and mobile devices. At the start of the questionnaire participants were informed of the purpose of the study, the confidentiality of their individual results, and the voluntary nature of their participation. Links to the questionnaire were distributed 3 months after the competitive event via email and social media. The assessments required approximately 20 min to complete.

The recall procedure adopted in this study addresses the limitations associated with using self‐reports to measure individual thoughts and emotional experiences before or during performance. Athletes can be reluctant to complete self‐reports during high‐level competitive events, as they may find the assessment invasive and distracting from their routine and preparation strategies (Harger & Raglin, 1994). Furthermore, actively attending to one's emotional responses can heighten awareness of debilitating symptoms linked to dysfunctional feelings, exacerbate their impact, and compromise performance. Therefore, a reliable retrospective assessment to capture thoughts and emotions at more convenient times can be a feasible alternative (Tenenbaum & Elran, 2003). Empirical evidence indicates that athletes with extensive competitive experience are able to accurately recall and describe thoughts and emotions experienced in past events (Hanin & Syrjä, 1996; Jokela & Hanin, 1999; Tenenbaum & Elran, 2003). We can assume that this is particularly true for high‐level competitive events, where the stakes are high.

#### 2.3.1 Data analysis

Before the main analysis, the mean total scores of the variables (i.e., Self‐confidence, Emotional arousal control, Worry, Concentration disruption, Challenge appraisal, Threat appraisal, Psychobiosocial experiences, and Self‐evaluated performance) were screened for the presence of univariate or multivariate outliers and possible violations to multivariate normality, linearity, and homoscedasticity (Hair et al., 2019). To test Hypothesis 1, we computed descriptive statistics and Pearson product‐moment correlation coefficients between variables. Correlation coefficients were interpreted according to Zhu's (2012) indications—namely, 0–0.19 = no correlation, 0.20–0.39 = low correlation, 0.40–0.59 = moderate correlation, 0.60–0.79 = moderately high correlation, and >0.80 = high correlation. Reliability of each scale was assessed using McDonald's omega (*ω*) values. A multivariate analysis of variance (MANOVA) was performed on the mean scores of the dependent variables to examine possible differences by gender.

**FIGURE 1:**
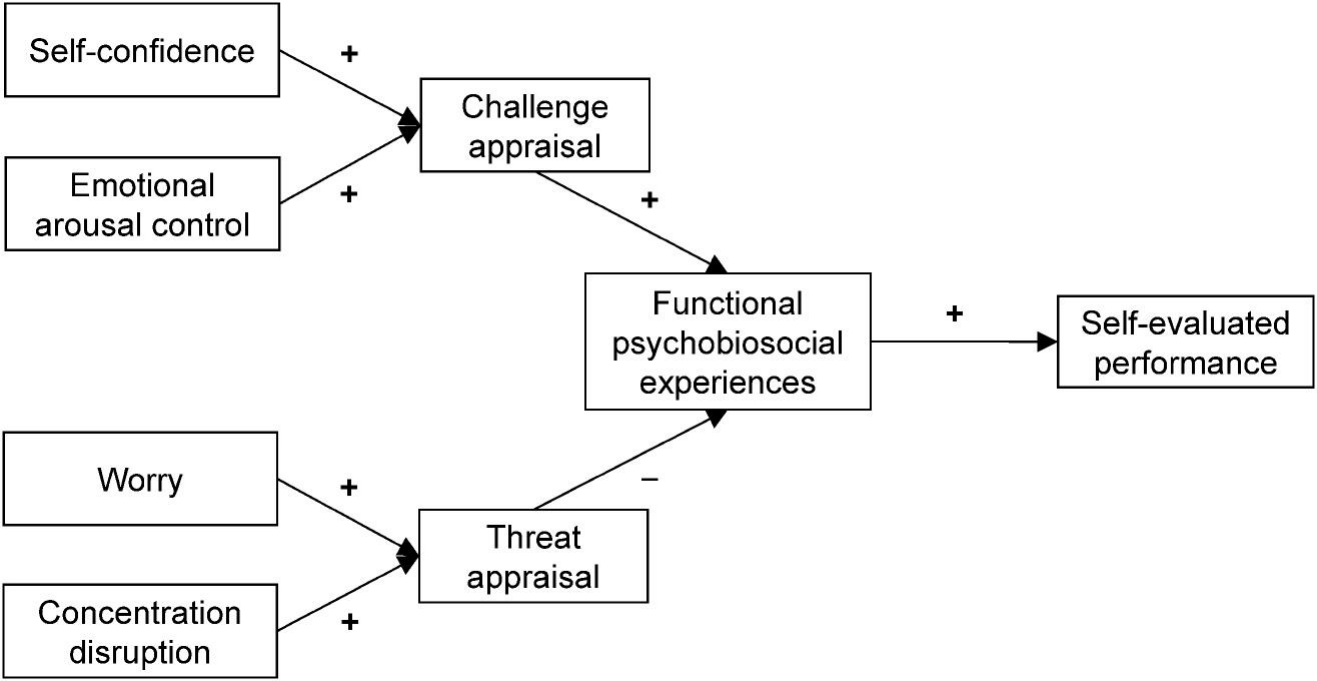
Hypothesized relationships among variables based on the multi‐states theory.

Path analysis in M*plus* version 8.5 was conducted to test Hypothesis 2 (see Figure 1). According to the rule of thumb proposed by several authors (Hair et al., 2019; Kline, 2016), at least 10 participants should be included per each estimated parameter. In the present study, we needed to estimate seven parameters, so the current sample size was appropriate. Good model fit was inferred with values of normed chi‐square (*χ* ^2^/df) smaller than 5, comparative fit index (CFI) and Tucker Lewis fit index (TLI) close to 0.95, root mean square error of approximation (RMSEA) and standardized root mean square residual (SRMR) smaller than 0.06 (Hu & Bentler, 1999). Indirect effects were assessed using the bias‐corrected bootstrap method based on 5000 resamples and 95% confidence intervals (CIs) around the standardized estimate (*β*). The indirect effect is assumed to be significant when its CI does not include zero (Hayes, 2022).

## 3 RESULTS

Six univariate outliers (*z* > |3.29|) were identified and then winsorized (Field, 2016) by replacing the top and bottom scores with the next highest or lowest value in the distribution, plus or minus 0.01. Mahalanobis' distances (*p* < 0.001) on winsorized data did not provide evidence for multivariate outliers. Assumptions of normality and multicollinearity were met. Reliability values were acceptable for all measures (see Table 1).

**TABLE 1:** Descriptive statistics, Pearson product‐moment correlation coefficients, and McDonald's omega (*ω*) values (*N* = 103).

Variables	*M*	*SD*	Skewness	Kurtosis	1	2	3	4	5	6	7	*ω*
1. Self‐confidence	4.820	0.769	−0.532	−0.245	––							0.799
2. Emotional arousal control	4.560	0.743	−0.317	−0.312	0.695^†^	––						0.847
3. Worry	2.879	1.098	0.640	−0.203	−0.348*	−0.250*	––					0.871
4. Concentration disruption	2.048	0.873	1.153	0.748	−0.327*	−0.158	0.590^§^	––				0.875
5. Challenge appraisal	6.021	0.800	−0.591	−0.595	0.286*	0.211*	−0.176	−0.266*	––			0.656
6. Threat appraisal	2.530	1.390	1.133	0.935	−0.185	0.025	0.589^§^	0.711^†^	−0.250*	––		0.840
7. Psychobiosocial experiences	2.584	0.739	−0.532	0.098	0.581^§^	0.515^§^	−0.299*	−0.262*	0.384*	−0.278*	––	0.927
8. Self‐evaluated performance	8.255	1.483	−0.679	0.884	0.326*	0.242*	−0.088	−0.227*	0.275*	−0.167	0.404^§^	0.887

*Note*: Correlation*low, ^§^moderate, ^†^moderately high (Zhu, 2012).

MANOVA showed significant differences by gender, Wilks' *λ* = 0.791, *F*(8, 94) = 3.096, *p* = 0.004, *η*_*p*_^2^ = 0.209. However, univariate follow up did not yield significant differences at *p* < 0.001, which was set to prevent type I error inflation due to multiple comparisons. In the whole sample, the mean scores of Self‐confidence, Emotional arousal control, and Challenge appraisal were higher than Worry, Concentration disruption, and Threat appraisal scores (all differences were significant at *p* < 0.001). The positive mean scores of Psychobiosocial experiences, indicating their functional effects on performance, were accompanied by high mean scores on Self‐evaluated performance. According to Hypothesis 1, all correlation coefficients between variables were in the expected direction (Table 1). In particular, Self‐confidence and Emotional arousal control were positively related to Challenge appraisal, Functional psychobiosocial experiences, and Self‐evaluated performance, while Worry and Concentration disruption were positively related to Threat appraisal, and negatively related to Functional psychobiosocial experiences. Concentration disruption was also negatively associated with Self‐evaluated performance.

**FIGURE 2:**
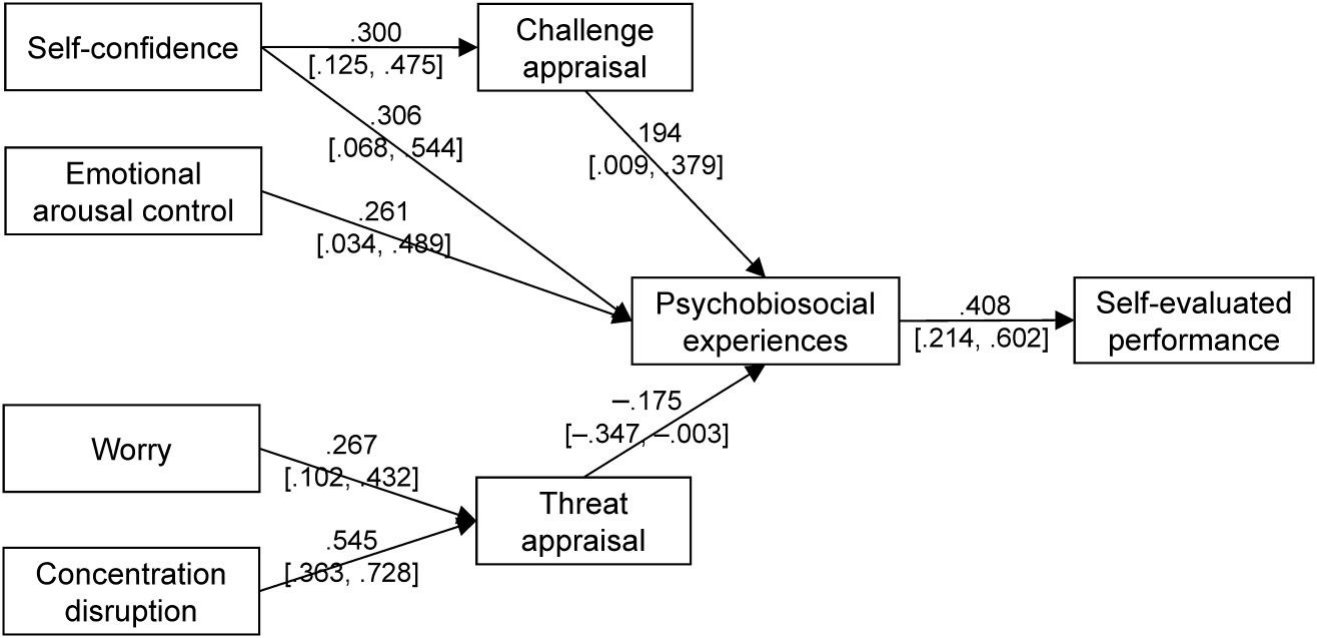
Path analysis results. All standardized values (*β*) are significant at *p* < .05 (95% CI are in square brackets).

Path analysis on the hypothesized model depicted in Figure 1 provided poor fit to the data, *χ* ^2^/df = 3.688, CFI =0.729, TLI = 0.602, RMSEA = 0.162 (90% CI = 0.117–0.208), SRMR = 0.148. Inspection of modification indices suggested adding two paths in the model from Self‐confidence and Emotional arousal control to Psychobiosocial experiences. The path from Emotional arousal control to Challenge appraisal was not significant and thus, removed from the model. The modified model represented in Figure 2 yielded acceptable fit, *χ* ^2^/df = 1.591, CFI = 0.955, TLI = 0.923, RMSEA = 0.076 (90% CI = 0.000–0.134), SRMR = 0.065.

As predicted in Hypothesis 2, findings showed: (a) a positive indirect effect from Self‐confidence to Performance via Challenge appraisal and Psychobiosocial experiences, *β* = 0.024, 95% CI = 0.002, 0.069; and (b) negative indirect effects from Worry, *β* = –0.019, 95% CI = –0.059, –0.003, and concentration disruption, *β* = –0.039, 95% CI = –0.094, –0.009, to Performance through Threat appraisal and Psychobiosocial experiences. We also observed a positive indirect effect from Emotional arousal control to Performance via Psychobiosocial experiences, *β* = 0.106, 95% CI = 0.021, 0.233.

## 4 DISCUSSION

Using the framework of the MuSt theory (Ruiz, Bortoli, & Robazza, 2021), the main purpose of the present study was to examine the multi‐dimensional relationships between self‐confidence, emotional arousal control, worry, concentration disruption, challenge and threat appraisals, psychobiosocial experiences, and self‐evaluated performance of elite kickboxers.

### 4.1 Relationships between variables

The first hypothesis of the study was confirmed. The positive individual trait‐like antecedents of feeling states (i.e., self‐confidence and emotional arousal control) were positively related to challenge appraisal, functional psychobiosocial experiences, and self‐evaluated performance, while negative trait‐like antecedents (i.e., worry and concentration disruption) were positively associated with threat appraisal and negatively related to functional psychobiosocial experiences and self‐evaluated performance (Table 1). Noteworthy, the mean scores of self‐confidence, emotional arousal control, and challenge appraisal in the whole sample were higher than the mean scores of worry, concentration disruption, and threat appraisal. Furthermore, the mean score of psychobiosocial experiences was positive, and therefore these experiences were perceived as functional to performance. Finally, the mean score of self‐evaluated performance was between “good” and “very good”.

Overall, the results suggest that the elite kickboxers perceived their psychophysical condition associated with competition as functional. Specifically, participants perceived themselves to be technically and tactically skillful, able to control their emotional arousal during competition, and able to maintain focus during the competitive event. The results are consistent with a large body of literature that clearly indicates that successful athletes commonly exhibit high self‐confidence, a strong performance focus, effective stress and distraction management, the ability to rebound from mistakes, an optimistic attitude, and emotional control (for reviews, see Jordet, 2015; Krane & Williams, 2021). In the present study, the kickboxers' dispositional characteristics associated with the perception of competition as a challenge rather than a threat were most likely precursors of functional psychobiosocial experiences and good performance.

### 4.2 Mediating role of competition appraisals and psychobiosocial states

The second hypothesis of the study was also supported. Indeed, path analysis showed a positive indirect link from self‐confidence to self‐evaluated performance via challenge appraisal and psychobiosocial experiences, as well as a negative indirect link from worry and concentration disruption to self‐evaluated performance via threat appraisal and psychobiosocial experiences (Figure 2). These results are consistent with the assumptions of the MuSt theory (Ruiz, Bortoli, & Robazza, 2021), and add to the existing evidence suggesting dynamic interactions between the individual, the task, and the environment in leading to individual appraisals and emotion‐related experiences, which can in turn impact the athlete's performance and wellbeing. The notion of challenge and threat appraisals is central in the MuSt theory as well as in other theoretical perspectives examining the appraisal‐emotion relationship (Blascovich, 2008; Lazarus, 2000; Sammy et al., 2021). Evaluating competitive demands as an opportunity to express one's resources (e.g., skills and abilities) can potentially lead to functional psychophysical states and high‐level performance, whereas seeing competition as potentially harmful due to task demands exceeding personal resources tends to lead to dysfunctional states and poor performance (Hase et al., 2019).

The results of the current study concur with previous study findings showing challenge appraisals to be related to more pleasant affect, better attentional control, and higher performance levels compared to threat appraisals (e.g., Brimmell et al., 2019; Wood et al., 2018). The results also extend the research examining the relationships among emotion‐related experiences. How kickboxers appraised their feelings and how they approached the competition was deemed to influence emotional and non‐emotional (i.e., psychobiosocial) manifestations of their subjective experiences and performance (Robazza et al., 2021). The mediating role of cognitive appraisals in the relationships between individual dispositions, psychobiosocial experiences, and performance suggests that the functionality level of these experiences and the resulting performance depend on both dispositional antecedents and the type of cognitive appraisal. Specifically, self‐confidence and emotional arousal control relate to athletes facing the event as a challenge. Challenge states are then linked to functional experiences and optimal performance. In contrast, worry and concentration disruption relate to athletes appraising the competition as a threat, which in turn, is associated with dysfunctional states and poor performance. In this view, cognitive appraisals would play a central role in explaining the observed relationships among the positive or negative individual dispositional antecedents, psychobiological experiences, and performance.

### 4.3 Strengths, limitations, and future research

This study contributes to the empirical research by examining key tenets of the MuSt theory, which aims to understand the dynamic interplay between individual dispositional characteristics, performance experiences, and athletic outcomes, as well as to identify effective self‐regulation strategies to help athletes deal with the demands of competition (Ruiz, Bortoli, & Robazza, 2021). The MuSt theory builds upon and extends established theoretical frameworks supported by substantial empirical evidence, such as the IZOF model (Hanin, 2000, 2007; Ruiz et al., 2017), the multi‐action plan model (MAP; Bortoli et al., 2012; Robazza et al., 2016), the cognitive‐motivational‐relational theory (Lazarus, 2000), and other competitive appraisal approaches (Blascovich, 2008; Meijen et al., 2020).

While our study provides insights into the relationship between dispositional factors, challenge and threat appraisals, psychobiosocial experiences, and performance outcomes in the context of combat sports, there are also limitations that should be acknowledged. Firstly, the reliance on athletes' ability to recall their experiences and knowledge of competition results may have influenced their reflections on feelings and performance. This may have been a potential bias associated with memory recall and perception of past performance, thereby affecting the accuracy of reported experiences and performance evaluations. Hence, caution should be taken when interpreting the results.

Although memory may have affected the accuracy of participants' responses, some variables in our study were dispositional (trait‐like), which are relatively stable over time. Moreover, given the repetitive nature of the sport and the high level of our sample, participants who routinely reflected on their competitive experiences were likely more aware and able to recall their pre‐event feelings accurately. However, future research should employ longitudinal designs to overcome the limitations of the cross‐sectional nature of the present study and the retrospective self‐evaluation of athletes, which may have introduced recall bias and limited the establishment of firm causal relationships between variables. Longitudinal designs are better suited for examining the temporal ordering of the relationships of the investigated variables. Additionally, experimental studies employing competitive pressure could provide a better understanding of the effects of dispositional variables on challenge and threat appraisals, psychobiosocial experiences, and performance.

It should also be considered that athletes retrospectively evaluated emotional experiences and cognitive appraisals by referring to their overall performance, potentially overlooking dynamic changes in situational variables over several events. Real‐time or longitudinal assessments could provide a more nuanced understanding of the dynamic interplay between the variables throughout different championship rounds. Moreover, levels of importance of the fight and familiarity with opponents may have influenced challenge and threat appraisals. Future studies should incorporate measures that capture these contextual variations to enhance the ecological validity of the findings.

Another limitation is that the athletes' positive performance evaluations may have been influenced by the fact that all participants had won a medal. To address this issue, future research should include participants from both winning and losing categories (or finalists and non‐qualifiers). Moreover, the relationship between challenge and threat appraisals and the performance of medalists raises an intriguing question regarding the potential influence of self‐expectations on self‐evaluated performance. For instance, threat appraisals combined with poorer self‐perceived performance of a medalist may reflect elevated expectations or high perfectionistic concerns. To address the latter and former issues and given that objective performance measures may not be suitable or easily implemented in combat sports, future studies could incorporate external performance evaluations, such as assessments from expert coaches, to complement athletes' performance evaluations.

Finally, future research should also examine the effects of a range of dispositional characteristics, as well as their interaction with environmental conditions (e.g., teammates, coach, parents), task features (e.g., individual and team sports, self‐paced and externally‐paced skills), and attentional mechanisms (e.g., action monitoring and voluntary control) on the process leading to performance and wellbeing of athletes, as advocated within the MuSt theory.

### 4.4 Conclusion and practical implications

Our findings, combined with those of previous studies (Ruiz, Luojumäki, et al., 2021, 2023), provide preliminary support to the multidimensional interplay between functional (i.e., self‐confidence and emotional arousal control) and dysfunctional (i.e., worry and concentration disruption) individual dispositions, challenge and threat appraisals, psychobiosocial experiences, and performance according to predictions derived from the MuSt theory. From an applied perspective, athletes should be encouraged to adopt self‐regulation procedures that can be applied across all stages of the performance process. Key strategies and techniques include self‐talk, which involves engaging in functional and constructive internal dialog (Fritsch et al., 2021); imagery, which entails mentally rehearsing successful performances, optimizing execution, and creating familiarity with competitive situations (Watt & Morris, 2021); cognitive restructuring, which focuses on identifying negative or irrational thoughts and replacing them with more positive and rational alternatives (Turner, 2016); mindfulness, which involves maintaining present‐moment awareness and staying attuned to the task (Fink & Ruiz, 2021); and action‐focused strategies, encompassing attending to and monitoring the core components of action (Bortoli et al., 2012; Robazza et al., 2016; Vitali et al., 2019). These strategies have been proven effective in helping athletes enhance self‐confidence, perceive competition as a challenge rather than a threat, experience functional emotions, facilitate action regulation, and improve their ability to manage competitive pressure.

## CONFLICT OF INTEREST STATEMENT

We certify that no party having a direct interest in the results of the research supporting this article has or will confer a benefit on us or on any organization with which we are associated. All authors declare that they have no conflict of interest.

## Data Availability

Data will be made available upon reasonable request to the corresponding author.
